# Virulent *Fusarium* isolates with diverse morphologies show similar invasion and colonization strategies in alfalfa

**DOI:** 10.3389/fpls.2024.1390069

**Published:** 2024-05-17

**Authors:** Jian Yang, Jing Han, Yuqing Jing, Siyang Li, Bo Lan, Qian Zhang, Kangquan Yin

**Affiliations:** ^1^ School of Grassland Science, Beijing Forestry University, Beijing, China; ^2^ College of Forestry, Beijing Forestry University, Beijing, China; ^3^ Lanzhou Institute of Husbandry and Pharmaceutical Science, Chinese Academy of Agricultural Science, Lanzhou, China

**Keywords:** fungal disease, root rot, forage crops, virulence, conidia, colonization

## Abstract

Root rot is a major disease that causes decline of alfalfa production, and *Fusarium* is a major pathogen associated with root rot. In this study, 13 *Fusarium* isolates were obtained from alfalfa with root rot in Gansu Province, the major alfalfa production region in China. The isolates were characterized by molecular genotyping (*ITS*, *TEF 1-α* and *RPB2* fragments) and identified as six species, which included the *F. acuminatum*, *F. incarnatum*, *F. oxysporum*, *F. proliferatum*, *F. redolens*, and *F*. *solani*. We found that their morphology varied significantly at both the macro- and micro-levels, even for those from the same species. We developed a low cost and fast pathogenicity test and revealed that all isolates were pathogenic to alfalfa with typical root rot symptoms such as leaf yellowing and brown lesions on the root and stem. However, the virulence of the isolates differed. We also found that the conidia of all isolates germinated as early as 24 hours post inoculation (hpi), while hyphae colonized the root extensively and invaded the xylem vessel by 48 hpi. Together our results reveal that different virulent *Fusarium* isolates use a similar invasion strategy in alfalfa. This natural plant-fungus pathosystem is intriguing and warrants further examination, particularly with regard to efforts aimed at mitigating the impact of multiple similar vascular pathogens on infected alfalfa plants.

## Introduction

Alfalfa (*Medicago sativa* L.) is known as the “queen of forage” with high nutritional value, rich in vitamins and protein, and can prevent soil erosion and fix nitrogen efficiently (Barnes et al., 1988; Veronesi et al., 2010; Li and Brummer, 2012). Therefore, alfalfa has a high feeding value and economic benefits (Guo et al., 2019). To date, alfalfa has been planted more than 40 million hectares worldwide (Zhang et al., 2010). However, owing to large-scale cultivation, alfalfa diseases have emerged (Wang et al., 2023). Among these, root rot disease is one of the most devastating threats to alfalfa, contributing to great production losses (Abbas et al., 2022). Root rot generally occurs in alfalfa cultivation areas, particularly in North America, Argentina, Russia, Australia, and Japan (Luo et al., 2019). In addition, root rot has been reported in various regions of China, such as Gansu, Xinjiang, and Heilongjiang provinces (Cai et al., 2021; Wang et al., 2021).

Root rot disease can be caused by various pathogens such as fungi, nematodes, and bacteria (Cormack, 1937; Heydari et al., 2012; Cao et al., 2020; Abbas et al., 2022). *Fusarium* spp. is one of the most prevalent pathogens (Cormack, 1937). Fusaria are soil-borne pathogens that can survive in soil and persist for a very long time, causing damage to many agricultural crops (Noble and Coventry, 2005). Furthermore, certain *Fusarium* species (for example, *F. culmorum* and *F. graminearum*) can produce trichothecene mycotoxins (e. g. DON), which not only inhibit germination, root growth, leaf mass of plants, seedling growth, and regeneration, but also exert toxic effects on animals, including feed refusal, induction of vomiting, growth retardation, increased susceptibility to infections, reduced ovarian function, and reproductive defects (Foremska et al., 1996; Rocha et al., 2005; Habler and Rychlik, 2016; Inbaia et al., 2023). In the reproductive history of *Fusarium* species, sexual and asexual spores act as propagules that initiate infection (Ajmal et al., 2023). However, far less than 20% of *Fusarium* species are sexually reproductive (Ohara and Tsuge, 2004). The asexual spores are generally called conidia, which are non-motile, walled, and haploid cells, and include three types: macroconidia, microconidia, and chlamydospores (Cole, 1986; Ohara and Tsuge, 2004). Macroconidia are usually sickle-shaped and microconidia are mostly oval or kidney-shaped. The chlamydospores were mostly spherical and thick-walled. Not all *Fusarium* spp. produce all forms of spores simultaneously (Ma et al., 2013). Conidia are critical in the life cycle of *Fusarium*, as they can protect the genome under adverse environmental conditions and serve as the primary means of dispersion (Osherov and May, 2001). During the infection process, the macroconidia and microconidia of *Fusarium* can attach to the surface of plant rhizomes and spread to other plants as secondary inocula and infectious agents (Rekah et al., 2000; Ohara and Tsuge, 2004). Chlamydospores are more durable survival structures in soil, more adaptable to adversity than macroconidia and microconidia, therefore are more contagious (Nicoli et al., 2013; Akhter et al., 2016). Taken together, conidia play a crucial role in the occurrence and circulation of root rot, and the quantity and growth rate of conidia also affect *Fusarium* infection and colonization.

Upon infection, *Fusarium* can damage the alfalfa root tip, leading to lesions of several colors: reddish, blackish, or brownish. The root system could then gradually become soft and decayed, accompanied by symptoms of severe chlorosis (Berg et al., 2017; Wang et al., 2023). At the same time, alfalfa grows slowly and eventually withers or even dies (Couture et al., 2002). And their pathogenicity changes seasonally (Cormack, 1937). However, the detailed infection strategy is unknown. Several methods have been reported for evaluating the pathogenicity of *Fusarium* strains. One of them is field evaluation, which mimics natural conditions (Miller-Garvin and Viands, 1994). The second method is a soil culture test conducted in a greenhouse or a growth chamber with inoculated soil (Li, 2002; Infantino et al., 2006; Liu, 2006). The third is the hydroponic screening method, which uses a liquid inoculum suspension in specially designed pots with alfalfa seedlings under growth chamber conditions (Cong et al., 2018). The current pathogenicity assays are either time-consuming or affected by microbial contamination, thus new methods are needed.

The aims of this study were (a) to identify pathogens associated with alfalfa root rot combined with morphological and phylogenetic analysis, (b) to establish a new method to evaluate the virulence of different isolates, and (c) to reveal the detailed infection strategy of *Fusarium* isolates in alfalfa.

## Materials and methods

### Plant materials and fungal isolates

**Figure 1:**
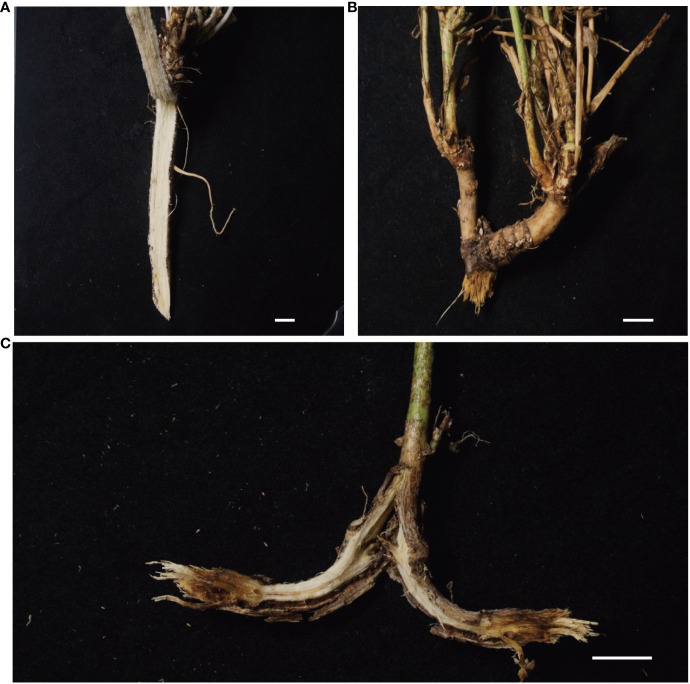
**(A–C)** Root rot symptoms in alfalfa grown in field. **(A)**, healthy root. **(B)**, brown lesions on the root and stem node of diseased alfalfa. **(C)**, Black streaking of the vascular system, showing necrotic symptoms. Bars = 1 cm.

Alfalfa plants with or without root rot symptoms were sampled from Dunhuang, Gansu, which is the major alfalfa production region in China (**Figures 1A–C**). The climate in this area is arid, with sufficient sunshine, large temperature differences between day and night (10–20°C), and low precipitation (40–200 mm/year). Diseased roots were harvested and washed free of soil using tap water. The roots were surface-sterilized with 70% ethanol for 10 s, followed by 1% sodium hypochlorite solution for 3 min. After surface sterilization, the roots were rinsed with sterile water three times and then cut into 1 cm long segments with disposable knife blades. To isolate fungi, we put four surface-sterilized alfalfa root segments onto each 9 cm petri dishes on potato dextrose agar (PDA) and incubated at 25°C in the dark for 5 days to allow fungal growth. A total of four PDA plates were used. Then we picked the emerging hyphae from the segments without bacteria contamination and transferred them to new PDA plates to purify single colony isolates.

### DNA extraction, PCR amplification, and sequencing

For DNA extraction, fungal cultures were grown on PDA for seven days. A small amount of mycelia was carefully collected from the single isolates using a sterile scalpel. Genomic DNA was extracted using the CTAB method (Richards et al., 1994). The PCR amplification was carried out for *TEF 1-α* fragment with the primer EF1 (5’-ATGGGTAAGGARGACAAGAC-3’) (O’Donnell et al., 2010) and the primer EF2 (5’-GGARGTACCAGTSATCATG-3’) (O’Donnell et al., 2010), for *RPB2* fragment with the primer 5f2 (5’-GGGGWGAYCAGAAGAAGGC -3’) (O’Donnell et al., 2010) and the primer 7cr (5’-CCCATRGCTTGYTTRCCCAT -3’) (O’Donnell et al., 2010) and for *ITS* rDNA with the primer ITS1-F (5’-CTTGGTCATTTAGAGGAAGTAA-3’) (Gardes and Bruns, 1993) and the primer ITS4 (5’-TCCTCCGCTTATTGATATGC-3’) (Vancov and Keen, 2009). The conditions for amplification of the *TEF 1-α* fragment were an initial denaturation step of 5 min at 95°C followed by 35 cycles of denaturation (95°C for 30 s), annealing (48°C for 30 s), and elongation (72°C for 30 s). The final elongation step was carried out at 72°C for 5 min. The annealing temperatures of the *RPB2* and *ITS* rDNA fragments were 52°C and 51.2°C, respectively. The remaining amplification conditions were the same as those for the *TEF 1*-*α* fragments. The PCR products were sequenced, and the obtained sequences were blasted against the NCBI database.

### Phylogenetic analysis

The nucleotide sequences obtained from Sanger sequencing were aligned using ClustalW (Thompson et al., 2003) and manually trimmed. The edited DNA Sequences of *TEF 1-α*, *RPB2*, and *ITS* rDNA were concatenated for phylogenetic analysis. We used the GTR + C + I default model of molecular evolution for maximum-likelihood (ML) analyses and bootstrapping with 1000 replicates, which were run using RAxML (Stamatakis, 2014). iTOL (ver3; http://itol.embl.de/) was used to visualize the phylogenetic tree.

### Virulence analysis

Alfalfa (cv. VISION) were used in virulence assays to evaluate the virulence of *Fusarium* isolates using a fast “unimpaired root dip inoculation on water agar” method at low cost. First, alfalfa seeds were surface-sterilized with ethanol for 15 s, followed by three washes with sterile water for 10 s. After removing the ethanol, the seeds were surface-sterilized in a 10% household bleach solution with 1% Triton-X for 15 min and washed three times with sterile water. Surface-sterilized seeds were sown on water agar medium (1% agar) and cultured in a growth chamber at 22°C under a 16 h light/8 h dark cycle. To prepare the inoculation, fungal conidia were collected from liquid potato dextrose cultures grown for five days by passing them through two layers of Miracloth (Millipore, Burlington, MA, USA). We measured conidia concentration under a light microscope (10× objective) using a hemacytometer, to which 10 μL of conidial solution was applied. A formula (conidia concentration = N/5 × 25 × 10^4^ per ml) was used to calculate conidia concentration. N: the total number of conidia in the five squares of the 25 grids zone (four corner squares and the middle square) (Yang et al., 2023). And the conidial concentration was adjusted to approximately 1 × 10^6^ per ml. An equal volume (10 μL) of fungal conidia was applied to each root tip of the alfalfa seedlings. Virulence of *Fusarium* was assessed by observing the degree of root decay. After inoculation of *Fusarium* conidia, root of seedling of alfalfa gradually developed lesions with different colors, including brown, yellow or red. Some root crowns would soften and rot and some plants showed chlorosis. We noticed that generally the degree of root decay stabilized after 4 weeks of inoculation. Thus, virulence was assessed by Diseased Grade four weeks after inoculation. Disease Grade was calculated to assess the severity of plant diseases and was classified on alfalfa roots as follows: Grade 1 “slight rotten” (0 to 25% area of root lesioned or rotted, the diseased symptoms were the slightest, Grade 2 “rotten” (25% to 50% area of root lesioned or rotted), Grade 3 “moderate rotten” (50% to 75% area of root lesioned or rotted), and Grade 4 “severe rotten” (75% to 100% area of root lesioned or rotted, and seedling wilted or died) (Xu et al., 2012; Wang et al., 2020). Virulence was assessed by visual inspection of the severity of the lesions. 30 alfalfa seedlings were inoculated in one experiment and repeated three times for each isolate.

### Histological observations

The *Fusarium* isolates were grown on PDA plates at 28–30°C until sporulation. We collected mycelia of each *Fusarium* isolate cultured on PDA medium, suspended them in ddH_2_O, and placed them under a microscope to observe the conidia of each *Fusarium* isolate.

Root sections inoculated for 24 h and 48 h were treated with 10% KOH for 45 min and neutralized with 2% HCL. After three washes with 1 × PBS, root sections were placed in staining solution (10 μg/ml WGA-fluorescein, 0.02% Tween20, 1 × PBS) overnight at 4°C and imaged using a Confocal Laser Scanning Microscope. And we performed z-stack processing with CLSM at 48 hpi to demonstrate that if hyphae inoculated the xylem. 3D visualization movies were undertaken to analyze microscopy images using Imaris software 9.2.0.

## Results

### Sample collection, fungi isolation and molecular identification

**Figure 2:**
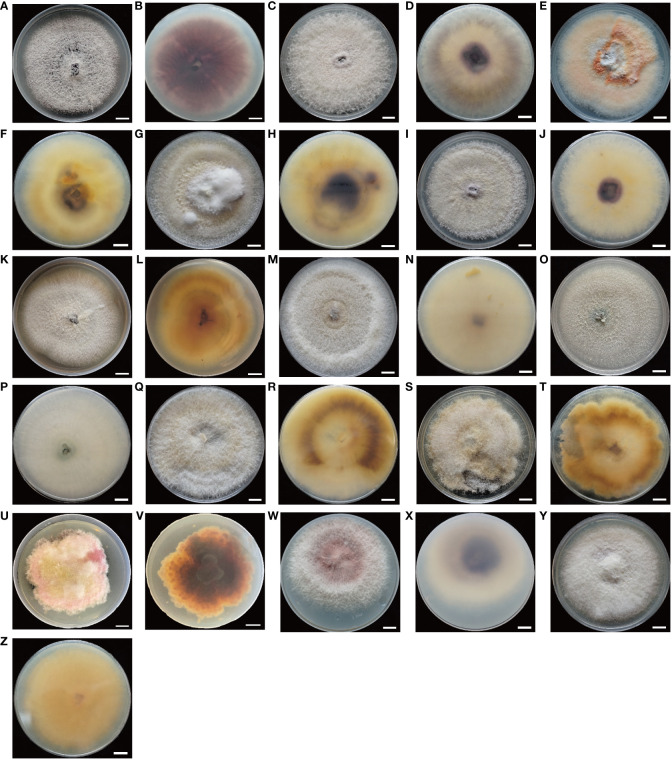
**(A–Z)**, morphological characteristics of *Fusarium* isolates. Colony’s upper and lower surfaces of *Fusarium* isolates grown on PDA incubated at 25 °C for two weeks. **(A, B)**:(1C), **(C, D)**:(2A), **(E, F)**:(2C), **(G, H)**:(3A), **(I, J)**:(3B), *F. proliferatum*; **(K, L)**:(1B), **(M, N)**:(2D), **(O, P)**:(3C), *F. solani*; **(Q, R)**:(1D), **(S, T)**:(2B), *F. incarnatum*; **(U, V)**:(1A), *F. acuminatum*; **(W, X)**:(2E), *F. oxysporum*; **(Y, Z)**:(3D), *F. redolens*. Bars = 1 cm.

**Figure 3:**
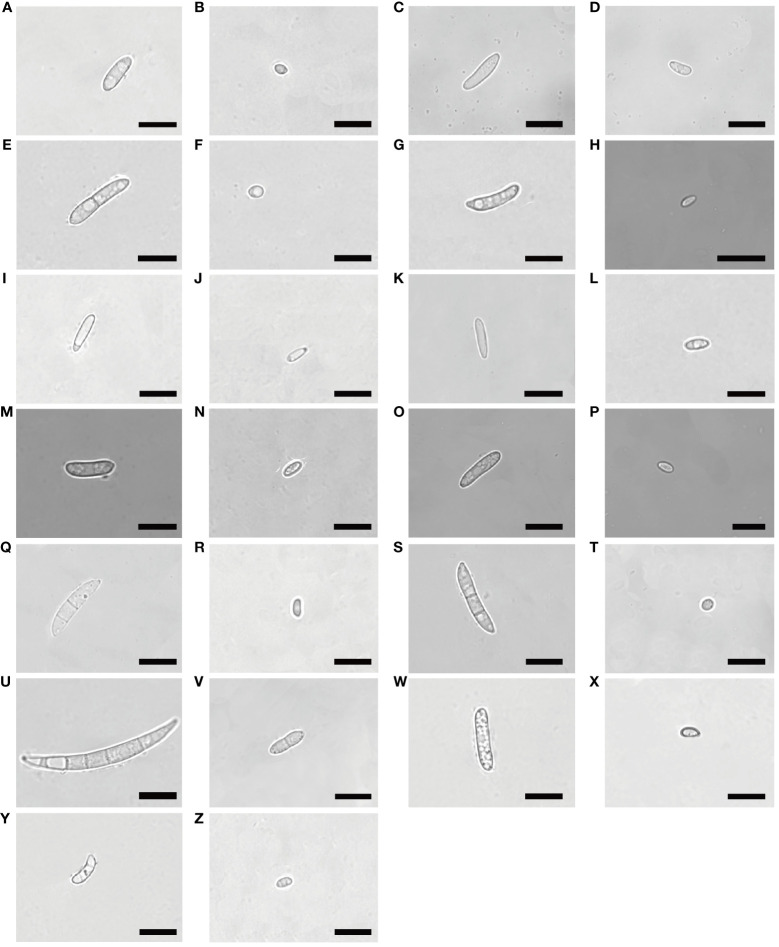
Morphological characteristics of macroconidia and microconidia of *Fusarium* isolates. Bars = 10 μm. **(A, B)**:(1C), **(C, D)**:(2A), **(E, F)**:(2C), **(G, H)**:(3A), **(I, J)**:(3B), *F. proliferatum*; **(K, L)**:(1B), **(M, N)**:(2D), **(O, P)**:(3C), *F. solani*; **(Q, R)**:(1D), **(S, T)**:(2B), *F. incarnatum*; **(U, V)**:(1A), *F. acuminatum*; **(W, X)**:(2E), *F. oxysporum*; **(Y, Z)**:(3D), *F. redolens*.

While the vascular tissue of the healthy root showed a white color (**Figure 1A**), the diseased root not only showed brown lesions outside but also dark brown discoloration inside the vascular tissue (**Figures 1B, C**). Diseased root tissues were plated on PDA plates for fungal isolation. Thirteen fungal isolates belonging to six species were identified from infected alfalfa roots based on three DNA barcodes (**Figures 2A–Z**). Of these, 1C (**Figures 2A, B**), 2A (**Figures 2C, D**), 2C (**Figures 2E, F**), 3A (**Figures 2G, H**) and 3B (**Figures 2I, J**) were *Fusarium proliferatum*; 1B (**Figures 2K, L**), 2D (**Figures 2M, N**), 3C (**Figures 2O, P**) were *Fusarium solani*; 1D (**Figures 2Q, R**), 2B (**Figures 2S, T**) were *Fusarium incarnatum*; 1A (**Figures 2U, V**) was *Fusarium acuminatum*; 2E (**Figures 2W, X**) was *Fusarium oxysporum*; 3D (**Figures 2Y, Z**) was *Fusarium redolens*. *F. proliferatum* was the most frequently isolated species (38.5%), followed by *F. solani* (23.1%) and *F. incarnatum* (15.4%).

### Morphological characterization of fungal isolates

**Figure 4:**
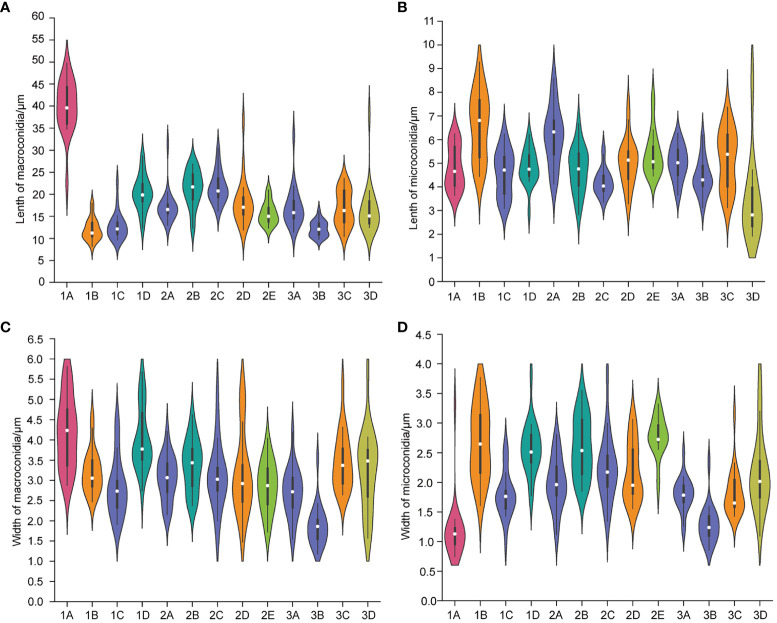
The length of macroconidia **(A)** and microconidia **(B)** and the width of macroconidia **(C)** and microconidia **(D)** of different *Fusarium* isolates. n = 20. (1A), *F. acuminatum*; (1B), (2D), (3C), *F. solani*; (1C), (2A), (2C), (3A), (3B), *F. proliferatum*; (1D), (2B), *F. incarnatum*; (2E), *F. oxysporum*; (3D), *F. redolens*.

The isolates on PDA were circular and formed cottony, and aerial hyphae on the surface. Morphology varied among the different isolates in terms of margin color, growth rate of hyphae, and shape of macroconidia and microconidia (**Figures 2**–**4**; **Supplementary Figure 1**).

#### F. proliferatum

The colony appeared cottony or floccose with abundant aerial mycelia (**Figures 2A–J**). The surface was initially white, but sometimes turned pale purple or orange with age and alternated dark purple or orange concentric rings on both the upper and lower surfaces, except 2C had yellow lower surface. The edges of the colonies were white or yellow in color. In addition, abundant sporulation was observed after 3 days of hyphal growth. Growth rate of mycelium was 10.810 ± 0.845 mm/day for 1C, 13.169 ± 0.861 mm/day for 2A, 7.053 ± 0.831 mm/day for 2C, 12.804 ± 0.427 mm/day for 3A, and 11.720 ± 0.403mm/day for 3B, respectively (**Supplementary Figure 1A**). Macroconidia were slender, fusiform, hyaline, with size ranging from 9.603–22.869 × 1.909–4.444 μm (mean ± SD = 12.844 ± 3.120 × 2.828 ± 0.718 μm) for 1C; reniform, slender, hyaline, blunt at both ends, with size ranging from 12.999–31.465 × 2.154–4.209 μm (mean ± SD = 17.223 ± 3.672 × 3.063 ± 0.541 μm) for 2A; straight, falciform, 1 septate, with size ranging from 16.173–30.449 × 1.560–5.231 μm (mean ± SD = 21.630 ± 3.535×3.113 ± 0.809 μm) for 2C; fusiform, 2 septate, with size ranging from 11.413–33.388×1.543–4.190 μm (mean ± SD = 17.146 ± 4.550 × 2.701 ± 0.598 μm) for 3A; fusiform, hyaline, blunt at both ends, with size ranging from 9.807–16.254 × 1.178–3.553 μm (mean ± SD = 12.187 ± 1.727 × 1.878 ± 0.505 μm) for 3B (**Figures 3A, C, E, G, I**, **4A, C**). Microconidia of 1C, 2A, 3A and 3B were reniform, hyaline and no septate, whereas 2C were oval and hyaline (**Figures 3B, D, F, H, J**). The size of microconidia were ranging from 3.090–6.240 × 0.987–2.599 μm (mean ± SD = 4.588 ± 0.943 × 1.797 ± 0.379 μm) for 1C, 4.121–8.595 × 1.117–2.807 μm (mean ± SD = 6.171 ± 1.216×1.989 ± 0.427 μm) for 2A, 3.647–6.273 × 1.192–2.511 μm (mean ± SD = 5.028 ± 0.712×1.795 ± 0.279 μm) for 3A, 3.109–6.201 × 0.852–2.339 μm (mean ± SD = 4.422 ± 0.754×1.288 ± 0.317 μm) for 3B, and 3.343–5.673 × 1.344–3.832 μm (mean ± SD = 4.208 ± 0.567×2.242 ± 0.545 μm) for 2C (**Figures 4B, D**).

#### F. solani

The colony appeared white to yellow with cottony mycelia (**Figures 2K–P**). Three isolates all had white concentric rings on the upper surfaces, but had different colors on the lower surfaces: 1B had the brown concentric rings (**Figures 2K, L**), 2D had the milky yellow concentric rings (**Figures 2M, N**) and 3C had the white concentric rings (**Figures 2O, P**). For *F. solani*, sporulation occurred after 3 days of mycelium growth. Growth rate of mycelium was 12.581 ± 0.380 mm/day for 1B, 11.019 ± 1.635 mm/day for 2D, and 12.381 ± 0.848 mm/day for 3C (**Supplementary Figure 1B**). Macroconidia were slender, straight to reniform, hyaline, blunt at both ends, with size ranging from 8.158–17.948 × 2.473–4.578 μm (mean ± SD = 12.032 ± 2.410 × 3.215 ± 0.530 μm) for 1B; slender, falciform, with tapering apexes and foot shaped bases, and size ranging from 11.873–36.633 × 1.471- 5.172 μm (mean ± SD = 18.214 ± 5.287×3.159 ± 1.002 μm) for 2D; falciform, hyaline, with size ranging from 10.378–23.590 × 2.646–5.362 μm (mean ± SD = 16.876 ± 3.950×3.465 ± 0.688 μm) for 3C (**Figures 3K, M, O**, **4A, C**). Microconidia were straight to reniform, with size ranging from 4.437–9.277 × 1.610–3.768 μm (mean ± SD = 6.599 ± 1.402 × 2.684 ± 0.609 μm) for 1B; ellipsoid, hyaline, with size ranging from 3.287–7.473 × 1.556–3.061 μm (mean ± SD = 5.045 ± 0.998×2.179 ± 0.485 μm) for 2D; reniform, hyaline, with size ranging from 3.122–7.377 × 1.428–3.160 μm (mean ± SD = 5.163 ± 1.225 × 1.831 ± 0.388 μm) for 3C (**Figures 3L, N, P**, **4B, D**).

#### F. incarnatum

Colony appearance was cottony or floccose with abundant aerial mycelia and was white with brownish yellow concentric rings on lower surface (**Figures 2Q–T**). The edge of mycelium was creamy yellow. The sporulation was observed after aerial mycelium growing 3 days. Growth rate of mycelium was 12.415 ± 1.382 mm/day for 1D and 9.909 ± 1.459 mm/day for 2B (**Supplementary Figure 1C**). The macroconidia were typical falciform shape, with tapering apexes and blunt bases, 2 septate, with size ranging from 12.298–28.730 × 2.739–5.676 μm (mean ± SD = 20.315 ± 3.913×4.028 ± 0.758 μm) for 1D; straight to falciform, 3 septate, blunt at both ends, with size ranging from 12.276–26.843 × 2.379–4.623 μm (mean ± SD = 21.360 ± 4.032 × 3.368 ± 0.626 μm) for 2B (**Figures 3Q, S**, **4A–C**). The microconidia were ellipsoid, hyaline, no septate, with size ranging from 2.935–6.216 × 1.775–3.824 μm (mean ± SD = 4.847 ± 0.719 × 2.581 ± 0.435 μm) for 1D; oval, hyaline, no septate, with size ranging from 2.820–6.687 × 1.851–3.549 μm (mean ± SD =4.733 ± 0.964 × 2.594 ± 0.503 μm) for 2B (**Figures 3R, T**, **4B, D**).

#### F. acuminatum

The colonies appeared cottony with abundant aerial hyphae (**Figures 2U, V**). The surface was pink to purple and the edge was yellow. Abundant sporulation was observed after aerial mycelium growth for 3 days. The growth rate of the mycelia was 8.325 ± 0.352 mm/day (**Supplementary Figure 1D**). Macroconidia were slender, straight to sickle-shaped with curved apexes and inconspicuous basal heels, 3- to 6- septate, with size ranging from 22.581–49.788 × 2.879–5.814 μm (mean ± SD = 40.030 ± 5.892×4.199 ± 0.868 μm) (**Figures 3U**, **4A, C**). Microconidia were oval or sickle-shaped with 1- to 2- septate, with tapering apexes and blunt bases, and with size ranging from 3.638–6.252 × 0.743–3.320 μm (mean ± SD = 4.822 ± 0.895×1.187 ± 0.519 μm) (**Figures 3V**; **4B, D**).

#### Fusarium oxysporum

The colony appeared cottony or floccose with abundant aerial mycelia, which varied in color from white to pale violet in concentric rings on both upper and lower surfaces (**Figures 2W, X**). The edges of the colonies were milky. The sporulation was observed after aerial mycelium growing 3 days. The growth rate of the mycelia was 12.750 ± 0.293 mm/day (**Supplementary Figure 1E**). Macroconidia were slender, straight to flat, 0- to 1- septate, with tapering apexes and foot shaped bases, and 12.309–20.978 × 1.724–4.040 μm (mean ± SD = 15.683 ± 2.468 × 2.854 ± 0.606 μm) (**Figures 3W**, **4A, C**). Microconidia were oval, ellipsoid or reniform, hyaline, with size ranging from 4.459–7.832 × 1.879–3.322 μm (mean ± SD = 5.425 ± 0.930×2.766 ± 0.334 μm) (**Figure 3X**; **Figures 4B, D**).

#### Fusarium redolens

Colony appearance was woolly or felt-shaped with abundant aerial mycelia. Mycelium were white to yellow with the passage of time and the edge was yellow (**Figures 2Y, Z**). *F. redolens* had white concentric rings on the upper surface, while showed yellow on the lower surface. The sporulation was observed after aerial mycelium growing 3 days. Growth rate of mycelium was 10.515 ± 0.691 mm/day (**Supplementary Figure 1F**). Macroconidia were straight to fusiform, with slightly blunt apexes and inconspicuous basal heels, with size ranging from 12.460–38.110 × 1.575–5.758 μm (mean ± SD = 16.585 ± 5.558×3.235 ± 0.980 μm) (**Figures 3Y**, **4A, C**). Microconidia were oval to ellipsoid, reniform, 0- to 1- septate, with size ranging from 1.917–9.062 × 1.096–3.922 μm (mean ± SD = 3.540 ± 1.847 × 2.181 ± 0.668 μm) (**Figures 3Z**, **4B, D**).

### Phylogenetic relationship among isolates of *Fusarium* spp. from alfalfa

**Figure 5:**
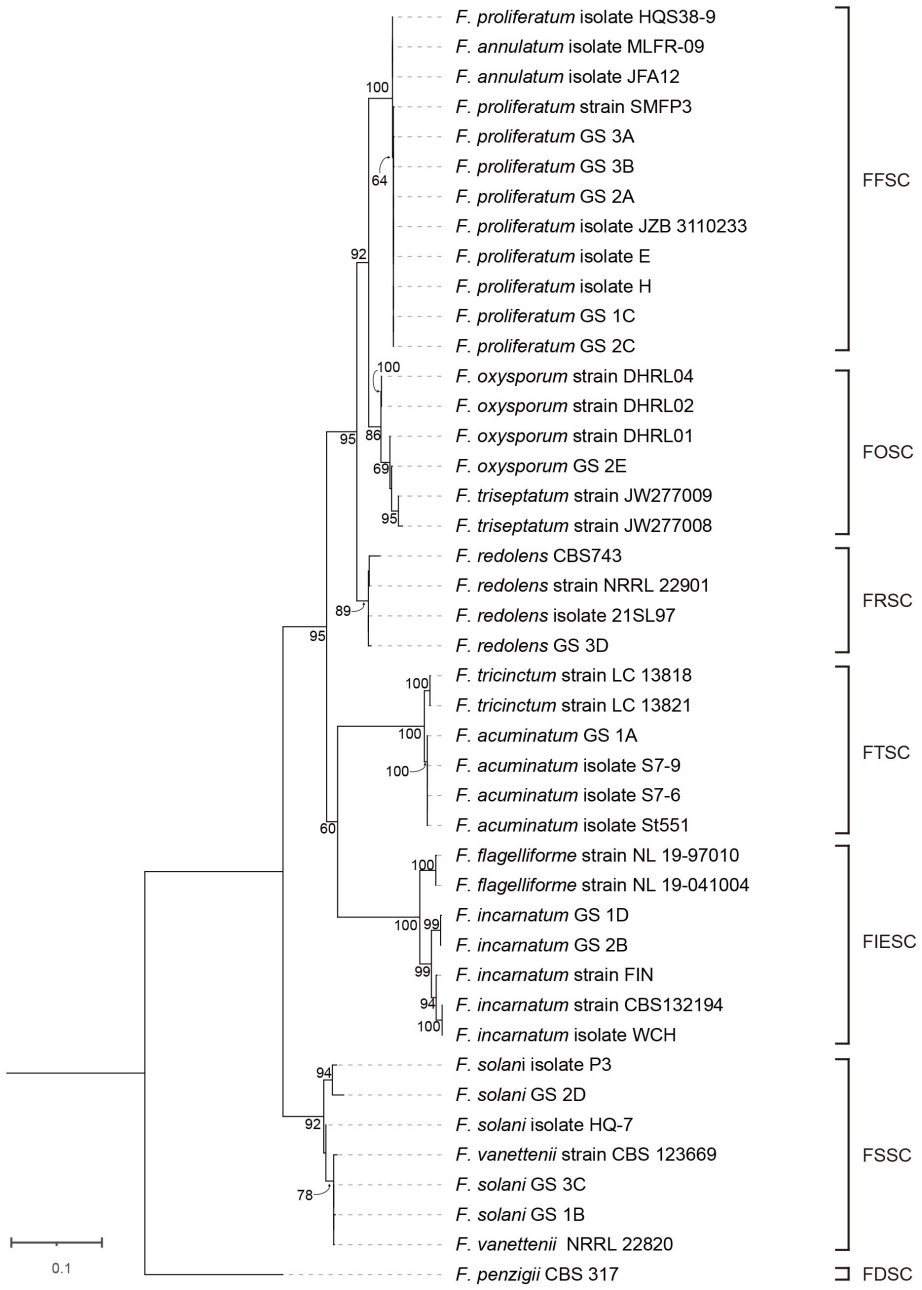
Phylogenetic tree of 13 *Fusarium* isolates from this study and 30 reference sequences from GenBank (**Supplementary Table 1**) based on the concatenated partial sequences of the *ITS*, *TEF 1-α* and *RPB2* genes. Letters indicate phylogenetic groups defined by 65% bootstrap support or more. The phylogenetic tree was inferred using the maximum likelihood method based on General Time Reversible + C + I model.

Forty-three Isolates from seven species complexes were chosen for phylogenetic study (**Supplementary Table 1**). As series of studies have demonstrated that multilocus DNA sequence other than single gene fragment should be used for accurately identifying and placing novel fusaria within a precise phylogenetic framework (O’Donnell et al., 2010), we used combined sequences of *ITS, TEF1-α, RPB2* genes to construct maximum-likelihood phylogenetic tree (**Supplementary Table 1**; **Figure 5**). These fragments have high resolution of *Fusarium* species (O’Donnell et al., 2010; Najafzadeh et al., 2020). For each isolate, we sequenced 361 bp for *ITS*, 333 bp for *TEF1*-*α*, and 399 bp for *RPB2*. The tree was rooted with *Fusarium penzigii* from the FDSC. Phylogenetic evolutionary relationships among the seven *Fusarium* species complexes were resolved by ML bootstrapping. These seven species complexes included a number of relevant *Fusarium* species: FFSC (n = 12), FOSC (n = 6), FRSC (n = 4), FTSC (n = 6), FIESC (n = 7), FSSC (n = 7) and FDSC (n = 1). Phylogenetic analyses of combined sequences of *ITS*, *EF1*-*α*, *RPB2* genes separated the 43 isolates into two distinct clades and six lineages, which corresponding to six species complexes. Clade A included FFSC, FOSC FRSC, FTSC, and FIESC. Clade B only included FSSC. We found strong bootstrap support for three nodes separating species complexes in the ML analysis: 92% ML bootstrap for node separating FFSC and FOSC, 95% ML bootstrap for node separating FFSC, FOSC and FRSC, and 95% ML bootstrap for node separating FFSC, FOSC FRSC, FTSC, and FIESC. In contrast, the FTSC received moderate bootstrap support (60%) as a sister to the FIESC.

There were eight nodes with bootstrap support of 100%, whereas 23 nodes with bootstrap support below 60%. We found a full bootstrap support for the node for separating three isolates (HQS38–9, MLFR-09, and JFA12) and other nine isolates of FFSC. However, the bootstrap for node separating these nine *F*. *proliferatum* isolates (SMFP3, 1C, 2A, 2C, 3A, 3B, E, H, and JZB3110233) was only 64%. Interestingly, all the five isolates (1C, 2A, 2C, 3A, and 3B) found in diseased alfalfa root in this study were less close to the above three isolates (HQS38–9, MLFR-09, and JFA12). In the FOSC lineage, there was a sister relationship between DHRL01 and other three strains (2E, JW277008, JW277009) with a moderate bootstrap support (69%). In the FRSC lineage, *F. redolens* isolate 3D clustered with 21SL97 and 22901 clustered with CBS743. In the FRSC lineage, 1A clustered with three isolates of *F. acuminatum* (St551, S7–6, and S7–9) with 100% support. In the FIESC lineage, *F. incarnatum* 1D clustered with 2B with strong bootstrap (99%). In the FSSC lineage, 2D was on the same branch with P3 with 94% bootstrap support, while 1B and 3C clustered with 22820 and 123699 with very close relationship.

### Virulence assay on seedlings under sterilized conditions

**Figure 6:**
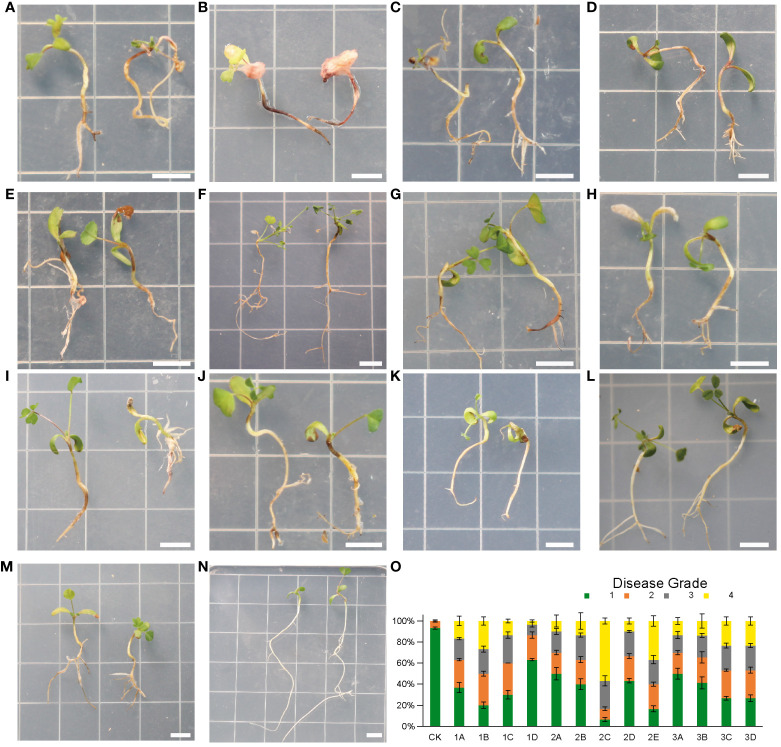
**(A–N)**, symptoms of alfalfa seedlings after inoculation with *Fusarium* isolates for 4 weeks. N(CK), healthy plants without fungal inoculation. **(A)** (1B), *F. solani*; **(B)** (2C), *F. proliferatum*; **(C)** (2E), *F. oxysporum*; **(D)** (1A), *F. acuminatum;* **(E)** (1C), G(3A), **(H)** (3B), *F. proliferatum*; F(2B), *F. incarnatum*; **(I)** (3C), *F. solani*; **(J)** (3D), *F. redolens;* **(K)** (1D), *F. incarnatum*; **(L)** (2A), *F. proliferatum*; **(M)** (2D), *F. solani*. **(O)**, Disease grades for infection assays of 13 *Fusarium* isolates at 4 weeks post inoculation. n = 30. Error bars indicated the SD of three biological replicates. Each small square has a side length of 1 cm. Bars = 0.5 cm.

To test whether each isolate was pathogenic to alfalfa, we established an easy, low cost and fast method using only agar in the recycled and autoclavable polypropylene boxes. To our surprise, all isolates we obtained from the diseased alfalfa root were pathogenic to alfalfa with typical root rot symptoms (**Figure 6**). However, severity differed in isolates in term of degrees of leaf yellowing, stunting, wilting and root brown lesion. Accordingly, we calculated the incidence rate of grade 4 for each isolate and we defined virulence into three groups based on the following criteria: if the incidence of grade 4 was above 25%, the isolate was grouped into the highly virulent group; if incidence of grade 4 was between 10% and 25%, the isolate was grouped into the moderately virulent group; if incidence of grade 4 was less than 10%, the isolate was grouped into the slightly virulent group (Koyyappurath et al., 2016) (**Figure 6**).

Isolate 1B, 2C and 2E were in the highly virulent group (**Figures 6A–C**). Among them 2C caused the most severe root rot symptom that root tips were blackened, while most of the rhizomes appeared lesions with several colors: blackish or brownish, or even turned reddish. In addition, all the root systems softened and rotted, and all leaves were yellowed and moldy. The disease incidence at grade 4 (severe rotten) of seedlings inoculation with 2C was more than 50% (**Figure 6O**).

Most of the isolates were placed in the moderately virulent group (**Figures 6D–J**), including 1A, 2B, 3C, 3D, and three isolates of *F. proliferatum* (1C, 3A, 3B). We found that about half of the root system showed brown lesions and leaves partially turned yellow.

1D, 2A and 2D were in the slightly virulent group (**Figures 6K–M**). Among them, 1D was the most slightly virulent isolate that causing far below 10% disease incidence at grade 4 (severe rotten) of seedlings after inoculation (**Figure 6O**). In addition, only a small portion of leaves of 1D showed yellowing symptoms, and the root system showed slight discoloration (**Figure 6K**).

### Proliferation and colonization of fungal isolates on and in alfalfa roots

**Figure 7:**
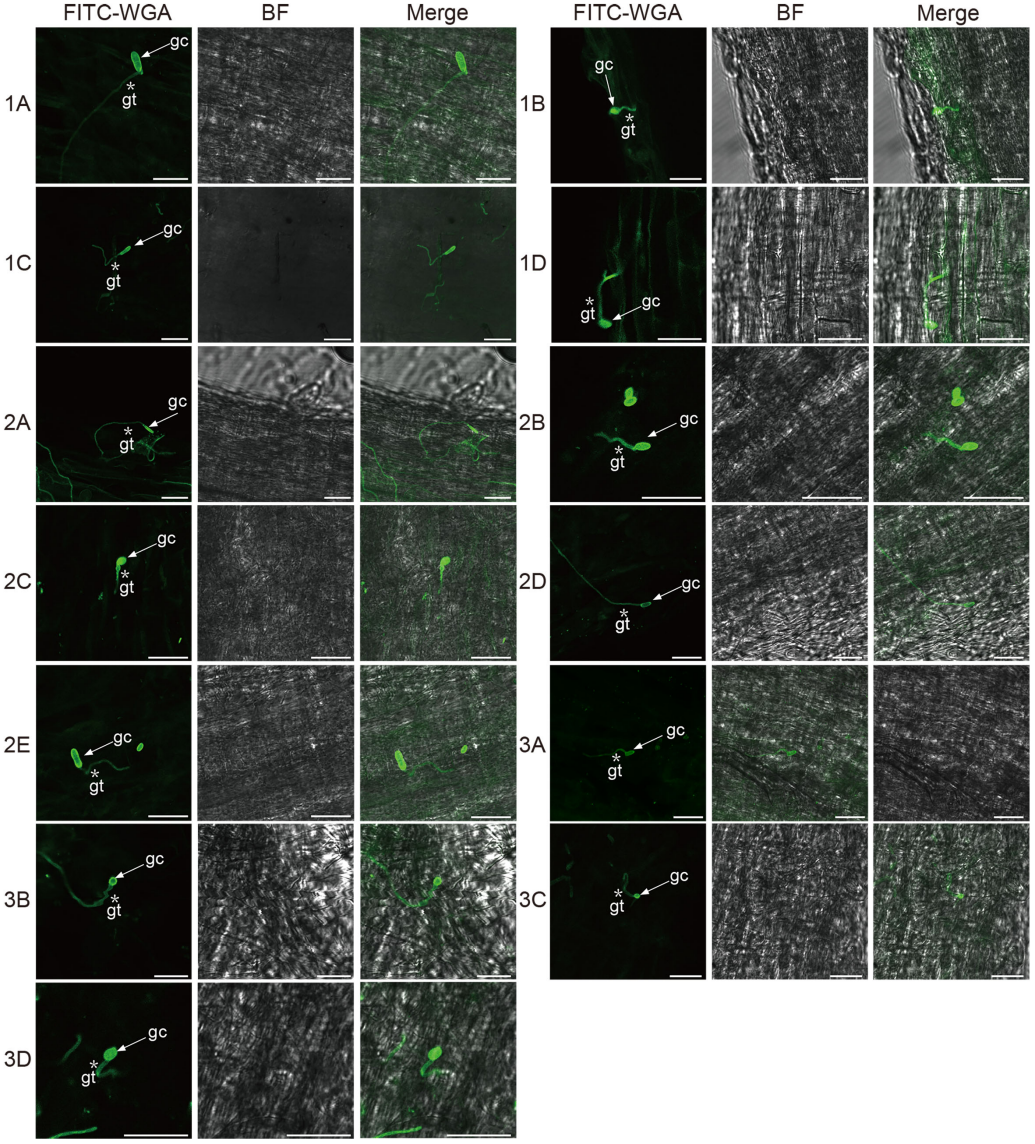
Confocal scanning laser microscopy images of root colonization by different *Fusarium* isolates on alfalfa roots at 24 hours post-inoculation. Arrows indicate germinated conidia and asterisks indicate the germ tubes of *Fusarium* isolates. Bar = 25 μm. (1A), *F. acuminatum*; (1B), (2D), (3C), *F. solani*; (1C), (2A), (2C), (3A), (3B), *F. proliferatum*; (1D), (2B), *F. incarnatum*; (2E), *F. oxysporum*; (3D), *F. redolens*.

**Figure 8:**
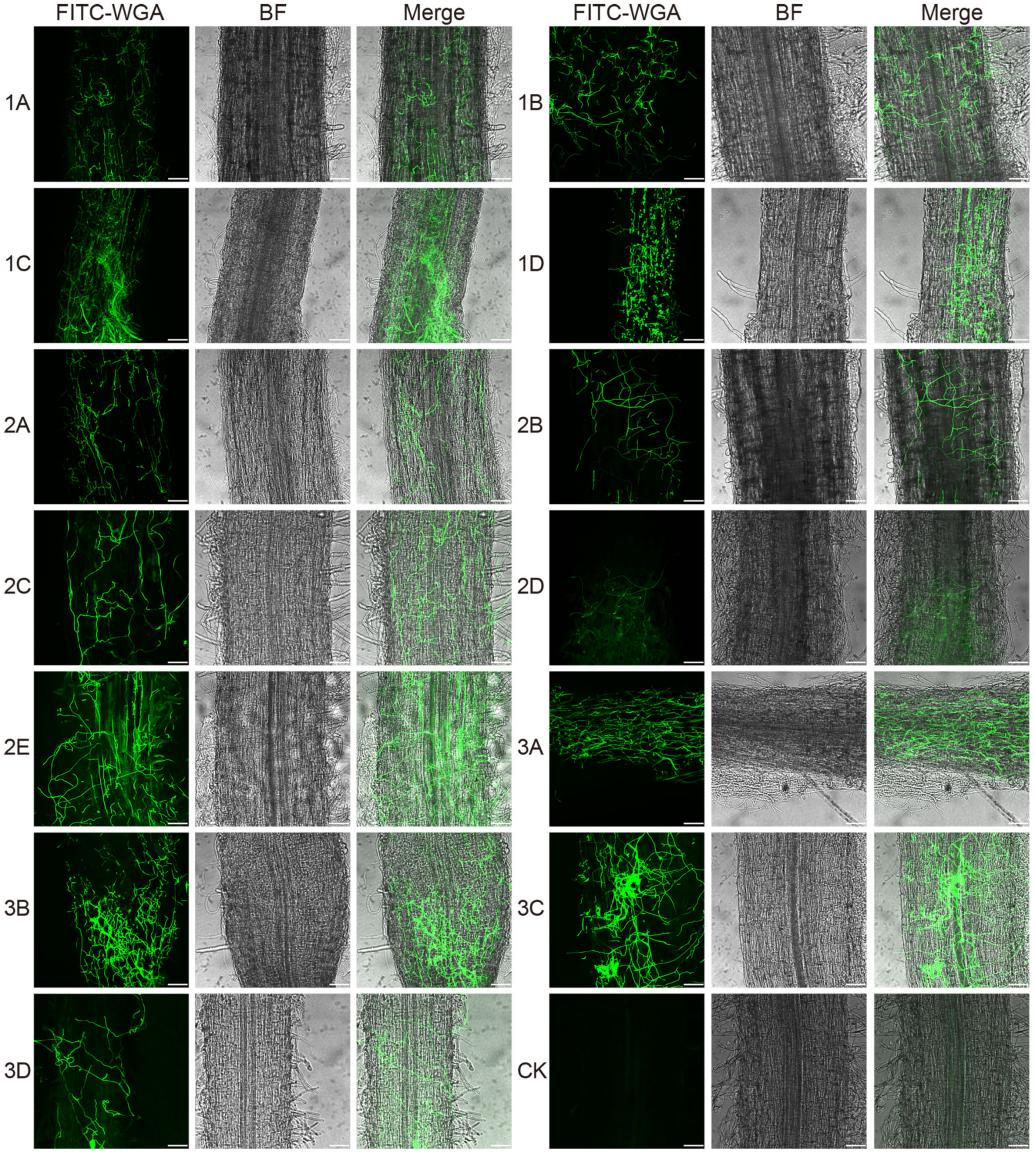
Confocal scanning laser microscopy images of root colonization by different *Fusarium* isolates on alfalfa roots at 48 hours post-inoculation. The images reveal a dense mass of hyphae covering the root surface. Bar = 75 μm. (1A), *F. acuminatum*; (1B), (2D), (3C), *F. solani*; (1C), (2A), (2C), (3A), (3B), *F. proliferatum*; (1D), (2B), *F. incarnatum*; (2E), *F. oxysporum*; (3D), *F. redolens*.

**Figure 9:**
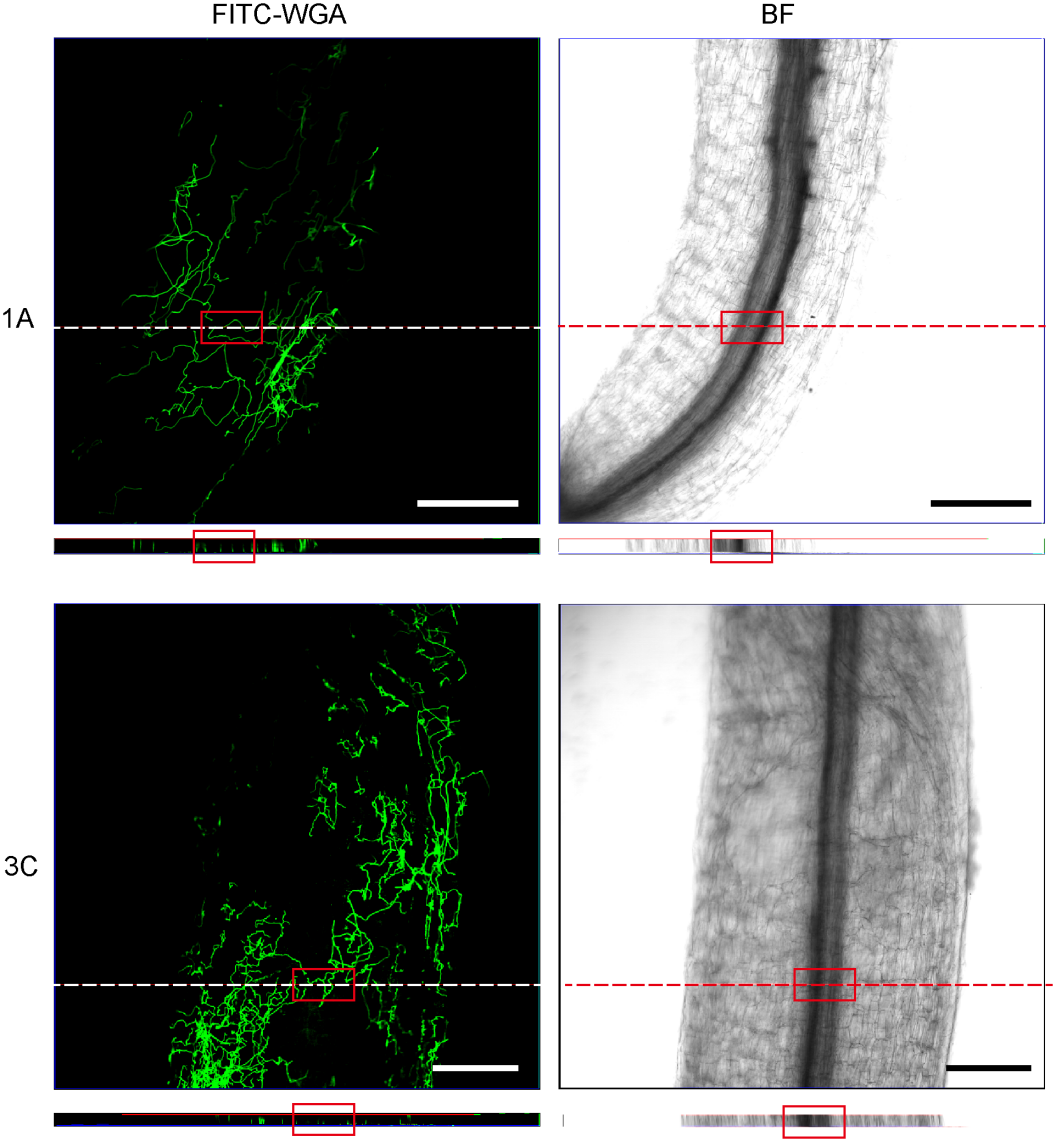
Confocal scanning laser microscopy z-stack images showed hyphae penetrating xylem at 48 hours post-inoculation. Orthogonal views were obtained from the areas indicated by white dotted line. The red square indicates that the hyphae reached the xylem. Bar = 200 μm. (1A), *F. acuminatum*; (3C), *F. solani*.

To investigate how *Fusarium* infect and colonize the alfalfa root, for each isolate, we used FITC-WGA staining and CLSM to observe the developmental processes associated with root infection. We found that the conidia of 13 *Fusarium* isolates germinated as early as 24 hpi, during which the bud tube emerged from the end of the conidia at the surface of root cells (**Figure 7**). Among them, conidia of isolates 1A, 2A, 2D, and 3B grew into hyphae in a fast fashion, resulting in long and slender hyphae (**Supplementary Figure 2**). Whereas the morphology of the germinated conidia of isolates 1B, 1C, 1D, 2B, 2C, 2E, 3A, 3C, and 3D was similar with tadpole shape, and their hyphae were shorter than above four isolates (**Supplementary Figure 2**). As shown by arrows in **Figure 7**, the shape of germinated conidia of isolates 1A, 1C, 2A, 2D, 2E, and 3A was flat and slender, and the shapes of other germinated conidia of isolates 1B, 1D, 2B, 2C, 3A, 3C, and 3D were round or oval (**Figure 7**). By 48 hpi, the hyphae were irregularly distributed in the root zone and successfully invaded the xylem (**Figure 8**). Among them, more hyphae of isolates 1C, 2B, 2C, 2E, 3A, 3B, and 3C were distributed on the xylem, and these hyphae were longer and densely clustered (**Figure 8**. 1C, 2B, 2C, 2E, 3A, 3B, 3C). Moreover, the hyphae of other isolates (1A, 1B, 2A, 3D) were shorter and some of which distributed on both sides of the xylem (**Figure 8**. 1A, 1B, 2A, 3D). In addition, several germinated conidia and some shorter hyphae of isolate 1D were observed at 48 hpi (**Figure 8**. 1D). Compared to other isolates, the number of hyphae in isolate 2D was the lowest (**Figure 8**. 2D). Moreover, we found the hyphae of these isolates had reached the xylem vessels through z-stack processing and 3D Visualization movies (**Figure 9**; **Supplementary Figures 3**, **4**, **Supplementary Movies 1**–**Supplementary Movie 3**).

## Discussion

The primary soil-borne fungi that cause alfalfa root rot worldwide are *Fusarium*, and thus most frequently isolated in rotted alfalfa root (Leath et al., 1971; Jiang et al., 2021). Interestingly, however, the composition of *Fusarium* species responsible for alfalfa root rot is highly diverse in different regions, probably due to heterogeneous environmental and geographical factors (Fang et al., 2019). In this study, we isolated six *Fusarium* species (*F. acuminatum*, *F. solani*, *F. proliferatum*, *F. incarnatum*, *F. oxysporum* and *F. redolens*) from diseased roots of alfalfa in Gansu province of China, which is one of the main production areas for alfalfa. Whereas in Inner Mongolia of China, 12 *Fusarium* species were identified with *F. acuminatum* most often isolated (Wang et al., 2023). In addition, it was reported that *F. avenaceum*, *F. oxysporum* and *F. solani* were most often isolated in northeastern USA (Salter et al., 1994). As in Africa, it was reported *F. oxysporum*, *F. acuminatum, F. semitectum*, *F. fusariodes* and *F. equiseti* were isolated from roots in main production areas in Egypt (Seif El-Nasar and Leath, 1983). These reports together with our current study demonstrate that alfalfa root rot is a disease complex (Leath et al., 1971) that can reduce yield in production areas, and also indicates the difficulties in managing multiple pathogens in the same time for an important perennial forage plant.

The morphological characters, including colony color, hyphae growth rate, conidia morphology etc., are crucial for accurate identification of *Fusarium* species (Summerell et al., 2003; Jiménez-Fernández et al., 2011). In this study, we found that each isolate we isolated from alfalfa diseased roots both had macroconidia and microconidia. However, the shape of their macroconidia was very diverse ranging from the representative sickle-shaped to straight, with length ranging from 8.158 μm to 49.788 μm. In contrast, the shape of their microconidia was mostly oval without septate, with length ranging from 1.917 μm to 9.277 μm. Our result was similar to that of pathogen characterization of post-flowering stalk rot in maize from agro-climatic zones of India, in which 71 isolates from four *Fusarium* species were identified (Harish et al., 2023). In addition, we found that the macroconidia from *F. acuminatum* were the largest with length up to 49.788 μm among our isolates. This was consistent with the findings of a comprehensive investigation of global isolates of *F. acuminatum*, in which the conidia produced by different isolates of *F. acuminatum* were variable in length with a range of 38.2 μm - 74 μm for 5-septate macroconidia, with some macroconidia up to 114.7 μm with 10 septa (Burgess et al., 1993). Although the aerial or plush mycelia can reach the edge of the PDA dish (9cm) within two weeks, their growth rate varied, even among isolates from the same species. The slowest isolate was 2C (*F. proliferatum*) with hyphae growth rate at 7.053 ± 0.831 mm/day. Whereas the fastest isolate was 2A (*F. proliferatum*) with hyphae growth rate at 13.169 ± 0.861 mm/day. We speculate that these variabilities in daily mycelial growth could be attributed to the presence of nutrient deficiencies (Robles-Carrión et al., 2016). In addition, these isolates showed different colors on PDA medium, even isolates from the same species showed different colors, not only for the upper but also for the lower surfaces of PDA plates. It is interesting to further explore the genetic mechanisms of pigmentation differences among isolates from the same species. The differences in conidia, hyphae growth rate and pigmentation of isolates within species also press the need of reliable molecular marker for accurate identification to species level (O’Donnell et al., 2010).

Molecular marker plays the important role during the species identification of *Fusarium* (O’Donnell et al., 2008). In this study, we used three markers (*ITS* rDNA, *TEF1-α*, *RPB2*) to identify fungi associated with alfalfa root rot disease. The combination of these three markers could recognize all isolates from six clades (FFSC, FOSC, FRSC, FTSC, FIESC, FSSC), but with varied bootstrap support. For example, we found strong bootstrap support (100%) for the FFSC, FTSC and FIESC clade, however the FOSC, FRSC and FSSC clades received 86–92% bootstrap support. Moreover, it is difficult to distinguish species within the same clade, such as *F. proliferatum* and *F. annulatum* in the FFSC clade, and *F. solani* and *F. vanetteni* in the FSSC clade. To better recognize *Fusarium* species in the same clade, combination of five gene datasets (*CaM*, *rpb1*, *rpb2*, *tef1*, and *tub2*) was suggested to recognize all species within the FFSC (Yilmaz et al., 2021). Whereas the combination of multi-locus dataset (*ITS*, *TEF-1α*, *CAM*, *RPB1*, *RPB2*) was suggested to recognize all species within FIESC clade (Wang et al., 2019). Recently, a total of 2020 strains isolated from diseased cereal crops were successfully identified to 43 species using multi-locus phylogeny, which including other new molecular barcodes (*CaM* and *H3*) (Han et al., 2023). Thus, it is a great challenge to discover universal barcodes for rapid and accurate identification of *Fusarium* species (O’Donnell et al., 2010; Schoch et al., 2012; Najafzadeh et al., 2020). As the pioneer research using 1001 homologous loci of 228 assembled genomes constructed a high-confidence *Fusarium* species tree (Han et al., 2023), we envision that multi-homologous loci based on whole genome sequencing will provide robust classifications with higher resolution.

The development of a rapid and reliable laboratory technique to assess the pathogenicity and virulence of numerous *Fusarium* strains on alfalfa would be extremely beneficial. In this study, we used sterilized water agar medium to evaluate the virulence of all the 13 *Fusarium* isolates on alfalfa seedlings. Our method has several advantages. First, sterilized condition excludes other microorganisms’ effect on virulence, which greatly improves the reproducibility of virulence assay. Second, as water agar provide no or limited carbon source for most bacteria, it can prevent growth of endophytic bacteria from alfalfa seeds, thus greatly reduces bacteria contamination. Third, our method can test the virulence in four weeks, which is faster than other methods with months (Miller-Garvin and Viands, 1994; Infantino et al., 2006). Therefore, our method has the potential to perform high-throughput virulence assay for *Fusarium* on alfalfa, with the possibility extending to other crop plants and even trees. In addition, this method can also be used to test pathogenicity alteration of complex infection which involves more than one isolates. Our research findings indicate that while all 13 *Fusarium* isolates were capable of infecting alfalfa, their virulence varied. It is worth noting that different isolates within the same species exhibit varying degrees of virulence. For example, in the *F. proliferatum* species, five isolates showed great virulent variations, which is in agreement with previous reports on *Fusarium* pathogens (Carter et al., 2002). We suspect that the variation in virulence among isolates may be attributed to the differential expression of genes related to virulence during the invasion of the isolates into alfalfa. Genetic diversity has been observed among isolates (Taylor et al., 2016), and it is possible that different isolates of the same species carry varying virulent genes and exhibit different level of virulence when interacting with host plants (Haapalainen et al., 2022). For instance, *SIX1*, the first avirulence gene discovered in *F. oxysporum*, is not present in all isolates, indicating the association of effector genes profile with virulence (Rep et al., 2004; Van Der Does et al., 2008; Lievens et al., 2009). Therefore, comparative study in virulent genes (effector gene identification, gene expression, etc.) among different isolates is promising for unravelling the mechanisms of pathogenicity variation in isolates.

The staining of 13 *Fusarium* isolates with a fluorescent dye revealed that the conidia of these isolates germinated and produced bud tubes within 24 hpi. Additionally, the hyphae of the isolates covered the alfalfa roots, and the *Fusarium* species penetrated the epidermal cells, further colonizing the xylem by 48 hpi. These findings suggest that the penetration of the 13 *Fusarium* isolates into the epidermis and xylem ducts of alfalfa roots occurred between 24 and 48 hours. Similar infection processes have been observed in other host plants, including conidial germination, bud tube emergence, and hyphal invasion of the xylem ducts (Zvirin et al., 2010; Guo et al., 2015). The nearly synchronized infection process of the 13 different isolates on the same host plants suggests that these isolates may have undergone concerted evolution in their interactions with the host plants in terms of infection initiation and colonization. This phenomenon also implies that different isolates may cooperate to quickly overcome the host plant’s defense system.

## Data availability statement

The datasets presented in this study can be found in online repositories. The names of the repository/repositories and accession number(s) can be found in the article/**Supplementary Material**.

## Author contributions

JY: Investigation, Writing – original draft, Writing – review & editing, Data curation, Validation, Formal Analysis, Visualization. JH: Data curation, Investigation, Validation, Writing – review & editing. YJ: Investigation, Writing – review & editing, Data curation, Software, Validation, Visualization. BL: Data curation, Software, Writing – review & editing. SL: Data curation, Investigation, Writing – review & editing. QZ: Resources, Writing – review & editing. KY: Resources, Writing – review & editing, Conceptualization, Funding acquisition, Investigation, Project administration, Supervision, Writing – original draft.

## Supplementary material

Supplementary Figure 1 The colony diameter of 13 isolates growing for different days. IBM SPSS statistics 24 (https://www.ibm.com/support/pages/downloading-ibm-spss-statistics-24) was used for statistical analysis, including average colony diameters and corresponding standard deviations. Error bars indicated the SD of three replicates. **(A)** (1C, 2A, 2C, 3A, 3B), *F. proliferatum*; **(B)** (1B, 2D, 3C), *F. solani*; **(C)** (1D, 2B), *F. incarnatum*; **(D)** (1A), *F. acuminatum*; **(E)** (2E), *F. oxysporum*; **(F)** (3D), *F. redolens*.

Supplementary Figure 2 The hyphae length of 13 isolates by 24 hpi. Error bars indicated the SD of at least three replicates. 1D, 2B, 3A (n = 6); 2C (n = 5); 3B (n = 4); eight other isolates (n = 3). (1A), *F. acuminatum*; (1B), (2D), (3C), *F. solani*; (1C), (2A), (2C), (3A), (3B), *F. proliferatum*; (1D), (2B), *F. incarnatum*; (2E), *F. oxysporum*; (3D), *F. redolens*.

Supplementary Figure 3 Confocal scanning laser microscopy z-stack images showed hyphae penetrated xylem at 48 hours post inoculation. Orthogonal views were made from areas indicated by white dotted line. The red square indicated the hyphae had reached the xylem. Bar = 200 μm. (1D), *F. incarnatum*; (3B), *F.proliferatum*.

Supplementary Figure 4 Confocal scanning laser microscopy z-stack images showed hyphae penetrated xylem at 48 hours post inoculation. Orthogonal views were made from areas indicated by white dotted line. The red square indicated the hyphae had reached the xylem. Bar = 200 μm. (2E), *F. oxysporum*; (3D), *F. redolens.*

Supplementary Table 1 Fungal isolates and GenBank accession numbers used in the phylogenetic analysis. *ITS* = internal transcribed spacer; *TEF1-α* = Elongation factor 1 alpha; *RPB2* = RNA polymerase IIsubunit.

Supplementary Movie 1 3D movie showed hyphae penetrated xylem at 48 hours post inoculation. Bar = 300 μm. 1D, *Fusarium incarnatum*.

Supplementary Movie 2 3D movie showed hyphae penetrated xylem at 48 hours post inoculation. Bar = 200 μm. 2A, *Fusarium proliferatum*.

Supplementary Movie 3 3D movie showed hyphae penetrated xylem at 48 hours post inoculation. Bar = 150 μm. 3C, *Fusarium solani*.
