# Supplementary figures and images for: Virulent Fusarium isolates with diverse morphologies show similar invasion and colonization strategies in alfalfa
Source: Front Plant Sci. 2024 May 17;15:1390069. doi: 10.3389/fpls.2024.1390069 (PMC11140090; doi:10.3389/fpls.2024.1390069)

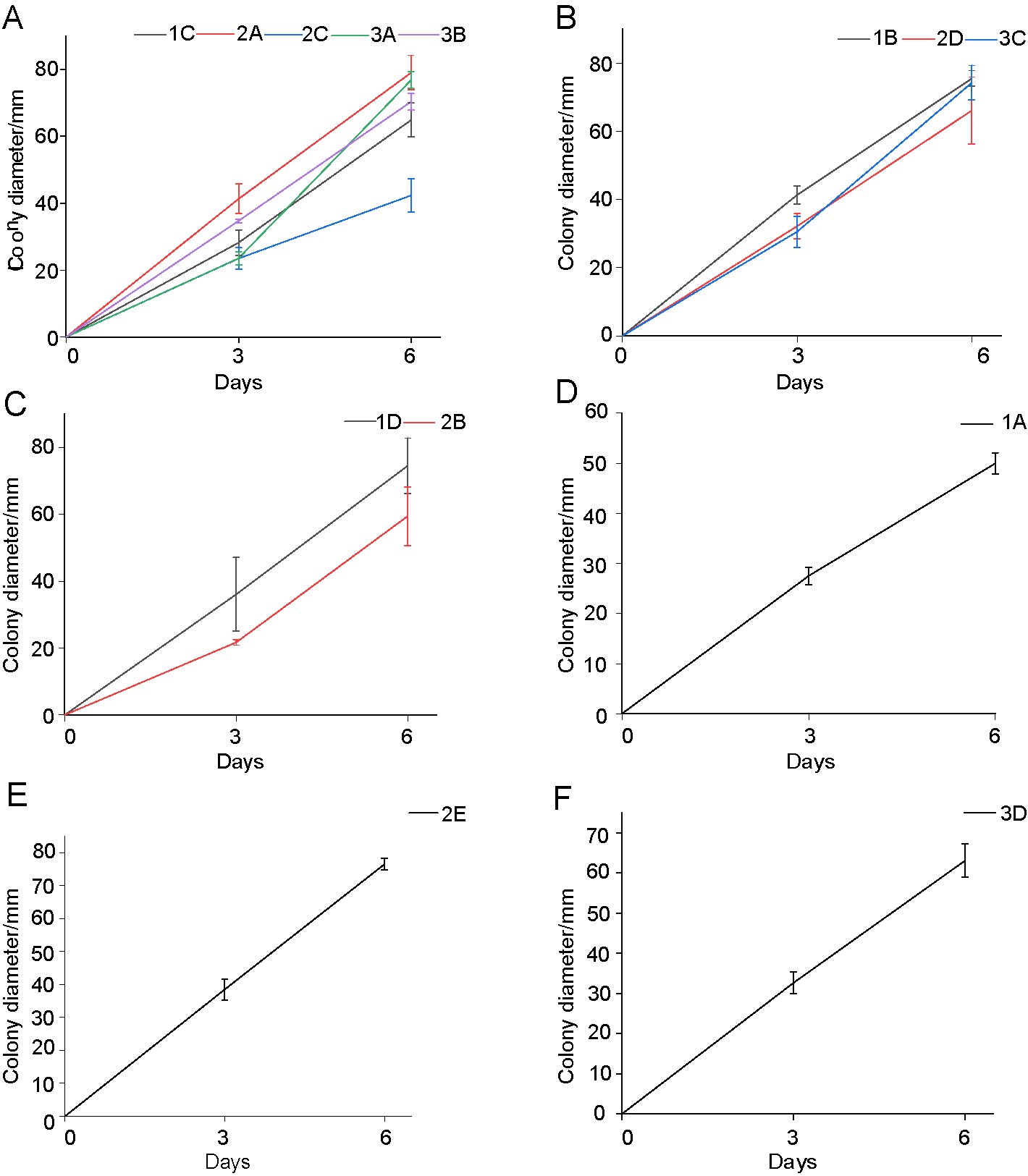

Supplement: Supplementary Figure 1 — The colony diameter of 13 isolates growing for different days. IBM SPSS statistics 24 (https://www.ibm.com/support/pages/downloading-ibm-spss-statistics-24) was used for statistical analysis, including average colony diameters and corresponding standard deviations. Error bars indicated the SD of three replicates. (A) (1C, 2A, 2C, 3A, 3B), F. proliferatum; (B) (1B, 2D, 3C), F. solani; (C) (1D, 2B), F. incarnatum; (D) (1A), F. acuminatum; (E) (2E), F. oxysporum; (F) (3D), F. redolens. [file Image_1.tif]

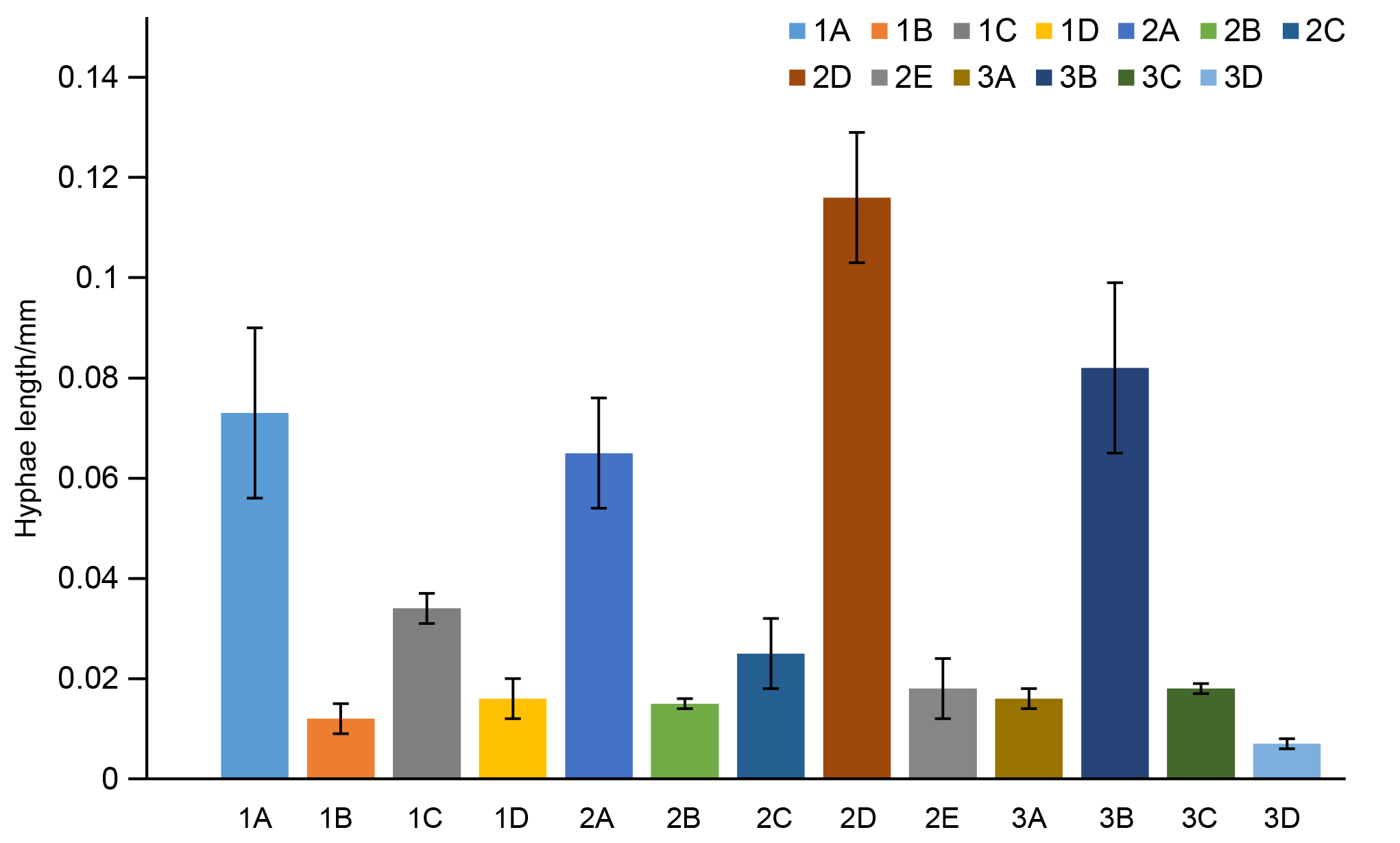

Supplement: Supplementary Figure 2 — The hyphae length of 13 isolates by 24 hpi. Error bars indicated the SD of at least three replicates. 1D, 2B, 3A (n = 6); 2C (n = 5); 3B (n = 4); eight other isolates (n = 3). (1A), F. acuminatum; (1B), (2D), (3C), F. solani; (1C), (2A), (2C), (3A), (3B), F. proliferatum; (1D), (2B), F. incarnatum; (2E), F. oxysporum; (3D), F. redolens. [file Image_2.tif]

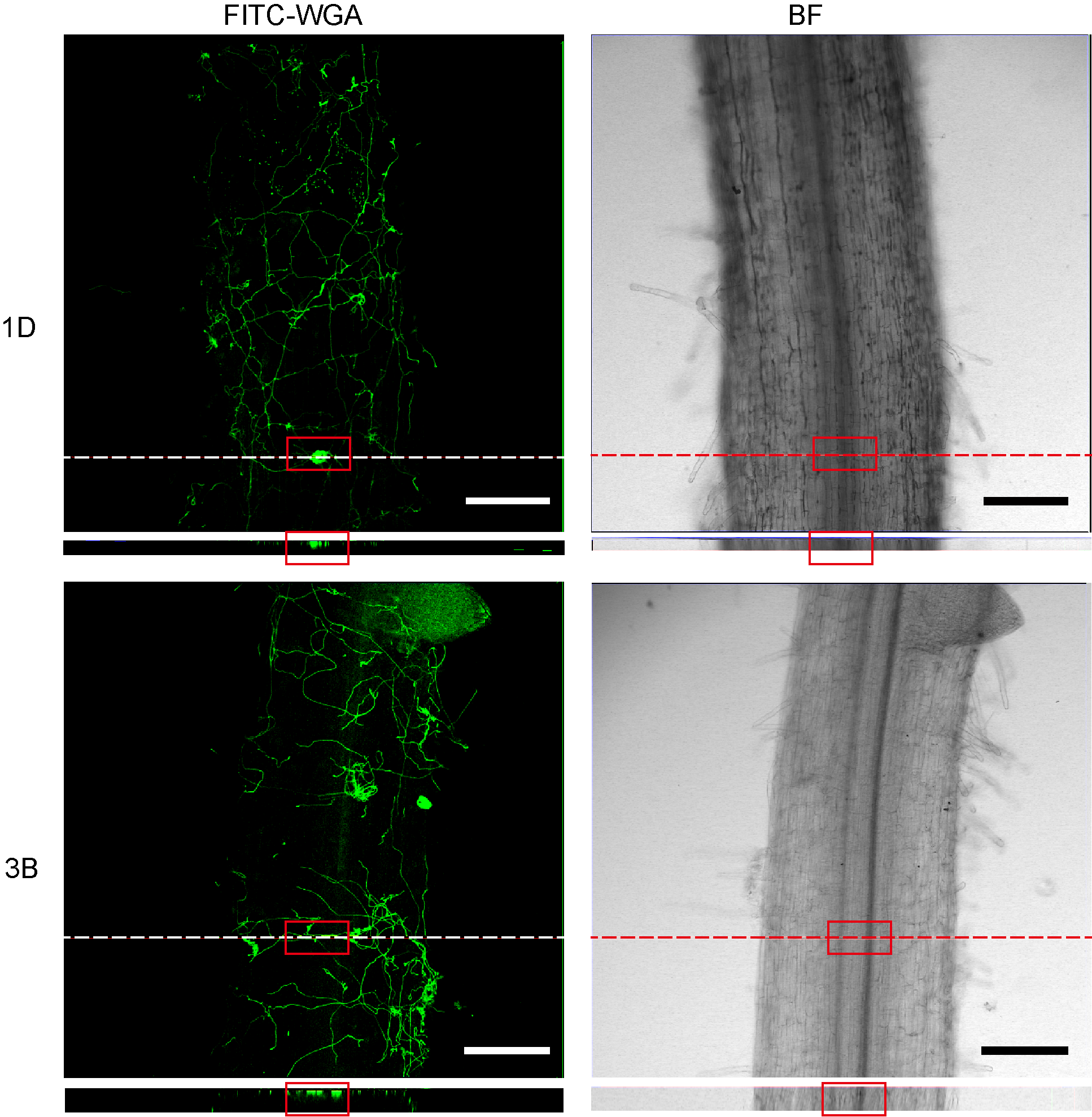

Supplement: Supplementary Figure 3 — Confocal scanning laser microscopy z-stack images showed hyphae penetrated xylem at 48 hours post inoculation. Orthogonal views were made from areas indicated by white dotted line. The red square indicated the hyphae had reached the xylem. Bar = 200 μm. (1D), F. incarnatum; (3B), F.proliferatum. [file Image_3.tif]

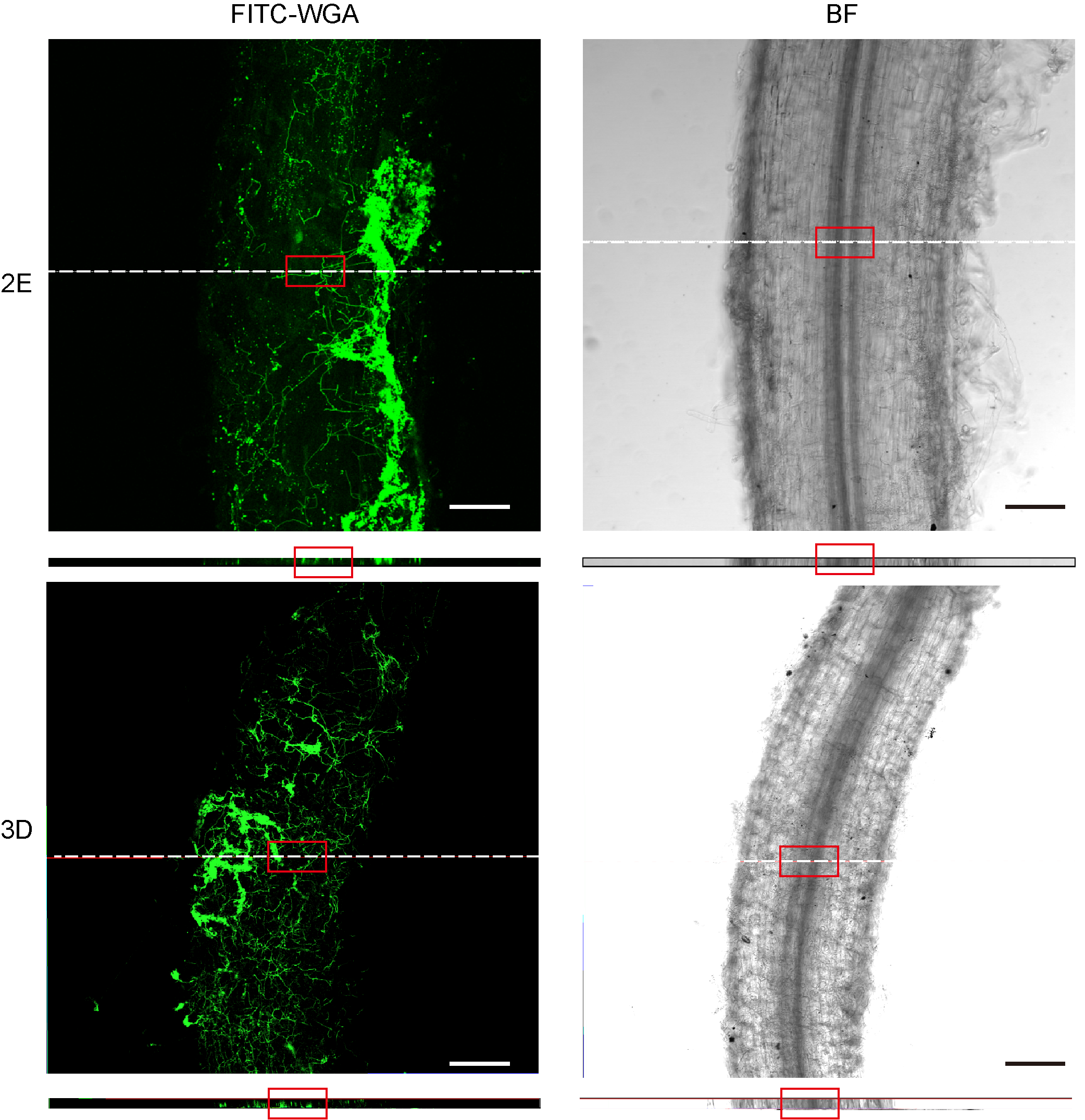

Supplement: Supplementary Figure 4 — Confocal scanning laser microscopy z-stack images showed hyphae penetrated xylem at 48 hours post inoculation. Orthogonal views were made from areas indicated by white dotted line. The red square indicated the hyphae had reached the xylem. Bar = 200 μm. (2E), F. oxysporum; (3D), F. redolens. [file Image_4.tif]
