# Supplementary material for: Virulent Fusarium isolates with diverse morphologies show similar invasion and colonization strategies in alfalfa
Source: Front Plant Sci. 2024 May 17;15:1390069. doi: 10.3389/fpls.2024.1390069 (PMC11140090; doi:10.3389/fpls.2024.1390069)
Supplement: Supplementary Table 1 — Fungal isolates and GenBank accession numbers used in the phylogenetic analysis. ITS = internal transcribed spacer; TEF1-α = Elongation factor 1 alpha; RPB2 = RNA polymerase IIsubunit. [file Table_1.docx]

**SUPPLEMENTARY TABLE 1**

| Species | Isolate | Host | Country | GenBank accession | | | Reference |
| --- | --- | --- | --- | --- | --- | --- | --- |
|  |  |  |  | *ITS* | *TEF1-α* | *RPB2* |  |
| *F. acuminatum* | S7-9 | Unknown | China | MZ057836 | MZ158155 | MZ158144 | Unknown |
| *F. acuminatum* | S7-6 | Unknown | China | MZ057835 | MZ158154 | MZ158143 | Unknown |
| *F. acuminatum* | St-551 | Unknown | China | MZ057834 | MZ158153 | MZ158142 | Unknown |
| *F. annulatum* | MLFR-09 | Cucumis melo var. cantalupensis | Spain | MZ355136 | OL960473 | OL944300 | Unknown |
| *F. annulatum* | JFA12 | Crocus sativus | China | MZ313130 | MZ338578 | MZ338573 | Mirghasempour et al.2022 |
| *F. flagelliforme* | NL 19-97010 | Soil | Netherlands | MZ890501 | MZ921844 | MZ921712 | Crous et al. 2021 |
| *F. flagelliforme* | NL 19-041004 | Soil | Netherlands | MZ890496 | MZ921839 | MZ921707 | Crous et al. 2021 |
| *F. incarnatum* | FIN | Nicotiana tabacum | China | ON226997 | ON256648 | OP650110 | Unknown |
| *F. incarnatum* | WCH | Actinidia chinensis Planch | China | ON391167 | ON456145 | ON456149 | Unknown |
| *F. incarnatum* | CBS132194 | Unknown | Netherlands | KF255427 | KF255470 | KF255542 | Unknown |
| *F. oxysporum* | DHRL-01 | Dendrobium chrysotoxum | China | MW599746 | MW703468 | MW703476 | Unknown |
| *F. oxysporum* | DHRL-02 | Dendrobium chrysotoxum | China | MW599747 | MW703469 | MW703477 | Unknown |
| *F. oxysporum* | DHRL-04 | Dendrobium chrysotoxum | China | MW599749 | MW703471 | MW703479 | Unknown |
| *F. proliferatum* | E | Onion | USA | MK299987 | MN684164 | MN653976 | Beck et al. 2019 |
| *F. proliferatum* | H | Onion | USA | MN685333 | MN684167 | MN653979 | Beck et al. 2019 |
| *F. proliferatum* | HQS38-9 | Unknown | China | OQ592804 | OQ633229 | OQ633400 | Unknown |
| *F. proliferatum* | JZB3110233 | Prunus avium | China | ON798832 | OP245125 | ON868386 | Unknown |
| *F. proliferatum* | SMFP3 | Salvia miltiorrhiza | China | MT371373 | MT371384 | MT934441 | Yang et al. 2020 |
| *F. penzigii* | CBS 317 | Unknown | UK | MH855543 | EU926324 | KM232362 | Unknown |
| *F. redolens* | NRRL_22901 | Unknown | USA | MT435063 | MT409452 | KU171708 | Gargouri et al. 2020 |
| *F. redolens* | 21SL97 | tobacco | China | OM666548 | OM622423 | ON237746 | Unknown |
| *F. redolens* | CBS743 | Allium fistulosum | Canada | AB304483 | MT010987 | MT010961 | Unknown |
| *F. solani* | HQ-7 | Unknown | China | MZ854208 | MZ997355 | MZ997364 | Unknown |
| *F. solani* | P3 | Unknown | China | MH900456 | MZ357258 | MZ357331 | Unknown |
| *F. triseptatum* | JW277009 | Soil | Netherlands | MZ890542 | MZ921889 | MZ921758 | Crous et al. 2021 |
| *F. triseptatum* | JW277008 | Soil | Netherlands | MZ890541 | MZ921888 | MZ921757 | Crous et al. 2021 |
| *F. tricinctum* | LC13818 | Acer palmatum | China | MW016692 | MW620153 | MW474678 | Unknown |
| *F. tricinctum* | LC13821 | Unknown | Japan | MW016695 | MW620156 | MW474681 | Unknown |
| *F. vanettenii* | CBS123669 | Unknown | Unknown | KM231796 | KM231925 | KM232364 | Lombard et al. 2015 |
| *F. vanettenii* | NRRL 22820 | Glycine max | USA | DQ094310 | AF178355 | EU329532 | O’Donnell K et al. 2008 |
| *F. acuminatum* | 1A | Alfalfa | China | OR591311 | OR666674 | OR666687 | Present study |
| *F. incarnatum* | 1D | Alfalfa | China | OR591314 | OR666677 | OR666690 | Present study |
| *F. incarnatum* | 2B | Alfalfa | China | OR591316 | OR666679 | OR666692 | Present study |
| *F. oxysporum* | 2E | Alfalfa | China | OR591319 | OR666682 | OR666695 | Present study |
| *F. proliferatum* | 1C | Alfalfa | China | OR591313 | OR666676 | OR666689 | Present study |
| *F. proliferatum* | 2A | Alfalfa | China | OR591315 | OR666678 | OR666691 | Present study |
| *F. proliferatum* | 2C | Alfalfa | China | OR591317 | OR666680 | OR666693 | Present study |
| *F. proliferatum* | 3A | Alfalfa | China | OR591320 | OR666683 | OR666696 | Present study |
| *F. proliferatum* | 3B | Alfalfa | China | OR591321 | OR666684 | OR666697 | Present study |
| *F. redolens* | 3D | Alfalfa | China | OR591323 | OR666686 | OR666699 | Present study |
| *F. solani* | 1B | Alfalfa | China | OR591312 | OR666675 | OR666688 | Present study |
| *F. solani* | 2D | Alfalfa | China | OR591318 | OR666681 | OR666694 | Present study |
| *F. solani* | 3C | Alfalfa | China | OR591322 | OR666685 | OR666698 | Present study |

**REFERENCE**

Beck, K. D., Reyes-Corral, C., Rodriguez-Rodriguez, M., May, C., Barnett, R., Thornton, M. K., et al. (2021). First report of *Fusarium proliferatum* causing necrotic leaf lesions and bulb rot on storage onion (*Allium cepa*) in southwestern Idaho. *Plant Dis*. 105(2), 494-494. doi: 10.1094/pdis-06-20-1399-pdn.

Crous, P. W., Hernández-Restrepo, M., van Iperen, A. L., Starink-Willemse, M., Sandoval-Denis, M., Groenewald, J. Z. (2021). Citizen science project reveals novel fusarioid fungi (*Nectriaceae, Sordariomycetes*) from urban soils. *Fungal Syst Evol*. 8(1), 101-127. doi: 10.3114/fuse.2021.08.09.

Gargouri, S., Balmas, V., Burgess, L., Paulitz, T., Laraba, I., Kim, H. S., et al. (2020). An endophyte of Macrochloa tenacissima (esparto or needle grass) from Tunisia is a novel species in the *Fusarium redolens* species complex. *Mycologia*. 112(4), 792-807. doi: 10.1080/00275514.2020.1767493.

Lombard, L., Van der Merwe, N. A., Groenewald, J. Z., Crous, P. W. (2015). Generic concepts in Nectriaceae. *Studies in Mycology*. 80(1), 189-245. doi: 10.1016/j.simyco.2014.12.002.

Mirghasempour, S. A., Studholme, D. J., Chen, W. L., Zhu, W. D., Mao, B. Z. (2022). Molecular and pathogenic characterization of *Fusarium* species associated with corm rot disease in saffron from China. *J Fungi*. 8(5), 515. doi: 10.3390/jof8050515.

O'Donnell, K., Sutton, D. A., Fothergill, A., McCarthy, D., Rinaldi, M. G., Brandt, M. E., et al. (2008). Molecular phylogenetic diversity, multilocus haplotype nomenclature, and in vitro antifungal resistance within the *Fusarium solani* species complex. *J Clin Microbiol*. 46(8), 2477-2490. doi: 10.1128/jcm.02371-07.

Yang, J., Wang, F., Wen, Y., Gao, S. X., Lu, C. T., Liu, Y. X., et al. (2020). First report of *Fusarium proliferatum* causing root rot disease in *Salvia miltiorrhizae* in China. *Plant Dis*. doi: 10.1094/pdis-09-20-1908-pdn.
